# “You Can't Always Get What You Want”: A Novel Research Paradigm to Explore the Relationship between Multiple Intentions and Behaviours

**DOI:** 10.1111/aphw.12071

**Published:** 2016-05-27

**Authors:** Falko F. Sniehotta, Justin Presseau, Julia Allan, Vera Araújo‐Soares

**Affiliations:** ^1^ Newcastle UniversityUK; ^2^ Fuse: The UK CRC Centre of Excellence for Translational Research in Public HealthNewcastle UniversityUK; ^3^ Ottawa UniversityCanada; ^4^ Aberdeen UniversityUK

**Keywords:** behaviour change, goal conflict, intention–behaviour gap, self‐regulation failure, social cognitive predictors of behaviour

## Abstract

**Objective:**

Research investigating cognitive moderators of the intention–behaviour relationship and psychological consequences of failure to enact intentions is usually conducted in a single‐behaviour paradigm. A multiple‐behaviour paradigm is introduced which overcomes bias inherent to single‐behaviour designs and allows testing of novel hypotheses. Two exploratory studies illustrate the utility of this new paradigm by investigating the role of cognitive predictors and psychological correlates of intention–behaviour relationships.

**Method:**

The proposed method involves measuring multiple intentions across common areas of life activity at baseline and corresponding behaviours at follow‐up. In two studies, 51 intentions and behaviours were assessed (49 by self‐report, 2 objectively). In Study 1, participants (*n *=* *126) also completed self‐reported measures of everyday cognitive failures and dysexecutive behaviours, crystallised intelligence (Mill Hill Vocabulary Scale) at baseline and Quality of Life (QoL; follow‐up). In Study 2, objective executive function measures (Stroop, Go/NoGo task and Word Fluency test) were completed by *N *=* *30 participants.

**Results:**

The total number of intentions, cognitive, and QoL measures were unrelated to the percentage of intentions enacted. Crystallised intelligence was related to successful intention implementation and problems with emotion regulation were associated with forming fewer intentions and with fewer *failed* intentions. QoL was strongly related with more intentions, regardless of whether or not these were implemented. Study 2 showed that cognitive flexibility (word fluency) and task errors, rather than Stroop effect and Go/No‐Go performance were related, to intention–behaviour congruence.

**Conclusion:**

Intention–behaviour relationships might be better understood when considering the multiple intentions and behaviours that people are engaged in at once at any one point in time. A multiple‐behaviour paradigm suggests novel hypotheses. Preliminary findings reported here require replication. Anticipated applications of the paradigm are outlined and discussed.

## Introduction

Traditional social cognition models of behaviour such as Ajzen's (1991) Theory of Planned Behaviour (TPB) hypothesise that intention is the most immediate predictor of behaviour, and various systematic reviews have shown that intention–behaviour relationships are indeed substantial (McEachan, Conner, Taylor, & Lawton, 2011). Intention is hypothesised to reflect a trade‐off between expectations about desirability and likelihood of achieving those expectations. Heckhausen described the process of forming an intention with the metaphor of crossing the Rubicon river, suggesting that once an intention has been formed, there is no way back; the only possible outcomes are success or failure (Heckhausen, 1991).

Over the last 20 years, a considerable gap between intention and behaviour has been noted (Orbell & Sheeran, 1998). At least half of all intentions are not enacted (Sheeran, 2002). This apparent gap has challenged the idea of wilful human behaviour held in traditional psychology. Recent evidence seems to suggest that humans are rather poor self‐regulators. Failures to implement intentions have been linked to difficulties in prospective memory (Einstein, McDaniel, Williford, Pagan, & Dismukes, 2003), limited willpower and processing capacity (Baumeister, Heatherton, & Tice, 1994) in the face of conflicting habits (Verplanken & Faes, 1999), distractions, temptations, and emotions (Hagger, Wood, Stiff, & Chatzisarantis, 2010; Metcalfe & Mischel, 1999). The pervasiveness of the intention–behaviour gap suggests an epidemic of behavioural self‐regulation failure and limited wilfulness of human behaviour (Ajzen, 2015; Sniehotta, Presseau, & Araújo‐Soares, 2014; Sniehotta, Presseau, & Araújo‐Soares, 2015).

### Cognitive Capacity as Moderator of the Intention–Behaviour Relationship

Cognitive abilities may explain, to some degree, discrepancies between intention and behaviour. Cognitive function measured in childhood predicts health behaviours that people choose to engage in over the lifespan (Gow, Corley, Starr, & Deary, 2012). This relationship is likely to reflect findings showing that intentionally engaging in healthy behaviours requires individuals to overcome short‐term costs in favour of long‐term gains (Adams, 2012; Hall & Fong, 2007). This kind of future‐focused, goal‐directed behaviour requires significant cognitive effort to implement and it has been argued that the successful implementation of an intentional health behaviour requires efficient higher‐level cognitive, or “executive”, function (Allan, Sniehotta, & Johnston, 2013; Hall & Fong, 2007). Individuals with weaker executive function and a tendency for cognitive failures are less likely to enact their intentions (Allan, Johnston, & Campbell, 2010, 2011; Hall, Fong, Epp, & Elias, 2008; McAuley et al., 2011). Correspondence between intention and diet/exercise behaviour is substantially greater for people with strong (than weak) executive function (Hall et al., 2008).

Executive functions represent the fluid mechanics of cognitive action control, i.e. they are the processes that allow effortful control of thoughts, emotions, and actions in response to changing contingencies. Successful goal pursuit also requires a different kind of cognitive ability: the pragmatic and strategic ability to identify relevant and realistic goals, and an awareness of how to develop, allocate, and apply the necessary means to achieve those goals. This cognitive ability is based on acquired knowledge about the self, one's resources, agency and the skills needed to conduct personal affairs (Baltes & Baltes, 1990; Freund & Baltes, 2002). These are based on cultural experiences and are often represented in measures of crystallised intelligence. It has been suggested that crystallised intelligence plays a critical role in the ability to select realistic goals which optimise what an individual can achieve with the available resources (Baltes, 1997).

### The Costs of Failure to Implement Intentions

The failure to enact intentions is thought to have costs in terms of wasteful investment of limited resources, resulting in frustration and reduced well‐being (Wrosch, Scheier, Carver, & Schulz, 2003). Cortisol and negative emotional responses to goal blockage have been shown in infants as young as 4–6 months (Lewis & Ramsay, 2005). Sustained commitment to failing goals has been linked to negative consequences (Baumeister & Scher, 1988; Brockner, 1992) and even to depression in adults (Metalsky, Halberstadt, & Abramson, 1987).

### From a Single‐Behaviour to a Multiple‐Behaviour Paradigm

Almost everything we know about how intention–behaviour relationships relate to cognitive abilities and measures of well‐being is based on studies looking at a single goal/intention and the resulting single behaviour. However, there is an increasing interest in developing the science of multiple behaviour change (Fleig et al., 2015; McSharry, Olander, & French, 2015; Nigg, Allegrante, & Ory, 2002) and to support such developments, innovations in theory and methodology are needed. There are considerable differences in how many goals people pursue, how difficult they are to achieve, how many resources one needs to invest in these goals, and how these goals relate to each other in terms of goal conflict, goal facilitation, and goal priority (Conner et al., in press; Emmons & King, 1988; Latham, Seijts, & Crim, 2008; Presseau, Francis, Campbell, & Sniehotta, 2011; Presseau, Sniehotta, Francis, & Gebhardt, 2010; Presseau, Tait, Johnston, Francis, & Sniehotta, 2013; Riediger & Freund, 2004).

Individuals with more cognitive resources may invest in forming more intentions or set themselves more challenging goals (Bandura, 1986). Social cognition models hypothesise that a given intention is a function of individual perceptions of desirability and the likelihood of achieving a behaviour. Thus, the increased likelihood of achieving based on the availability of better cognitive resources might lead to those with better cognitive resources forming more intentions. Studies focusing on a single behaviour are unlikely to provide an unbiased assessment of how intention–behaviour congruence relates to hypothesised cognitive predictors or affective consequences as they do not account for the various additional intentions individuals are likely to be pursuing that require investment of resources, facilitate or hinder implementation of other intentions, and provide additional sources of failure and opportunities for success.

When considering only studies that focus on a single behaviour at a time, we are potentially left with a biased estimation of the pervasiveness of intention–behaviour gaps. What are people doing, if not enacting the single intention of interest in a given study? Considering a given intention–behaviour gap alongside the other behaviours competing for available resources might improve our understanding of the enactment of a given intention.

The aim of this manuscript is to introduce a new research paradigm that could stimulate novel research and improve the understanding of the intention–behaviour relation, its predictors and consequences. Within this paradigm the intention–behaviour gap can be assessed for each individual across an array of behaviours. Successful enactment of intentions can be related to the total number of intentions pursued. Use of this paradigm is illustrated with two initial proof‐of‐principle studies addressing four main research questions:

1. How many behaviours do individuals intend to enact at any one time?
2. To what extent does the number of intentions generated relate to overall success and failure in enacting these behaviours?
3. To what extent does executive function (fluid cognitive resources) and crystallised intelligence (static cognitive ability) relate to the number of intended behaviours, and overall success and failure in intention enactment?
4. To what extent does the number of intended behaviours, and overall success and failure in enacting them, relate to QoL and depression?

## Study 1

To date it is not known how the number of intentions individuals form relates to the number and proportion of successfully implemented intentions. Study 1 tests four hypotheses:

1. Forming too many intentions may over‐commit individual resources and lead to self‐regulation failure. The more intentions people form, the lower the proportion of intentions they will successfully implement.
2. Individuals with higher levels of crystallised intelligence will form more intentions that they *successfully* enact.
3. People with better *executive functioning* (fewer cognitive failures) will translate a higher proportion of their intentions into behaviour.
4. People who have more failed intentions will have lower levels of well‐being and higher levels of depression.

## Methods Study 1

### Design

Study 1 was a prospective survey. Self‐reported intentions, demographics, behaviour, crystallised intelligence, and executive function were taken at baseline via an online questionnaire during autumn term in a UK university. Participants were followed up one week later with a measure of their behaviour and QoL (including well‐being and depression).

### Participants

*N *=* *126 students (*n *=* *97 female; 77%; mean age = 24.5, *SD* = 8.2) provided digital informed consent via a website and completed the baseline questionnaire; *n *=* *116 (91%) completed the follow‐up one week later.

### The All‐Intentions Method (AIM)

All intention items from 185 original papers included in a systematic review of the TPB were extracted (Armitage & Conner, 2001). In addition, intention items for 30 behaviours from a study by Norman, Sheeran, and Orbell (2003) were added. Duplicates and items that were not applicable to the general adult population in the UK (e.g. professional behaviours) or not relevant for a follow‐up period of one week (e.g. attending annual health checks) were excluded, leaving 51 eligible behaviours (see Appendix S1). Intention items for the 51 behaviours were adjusted to correspond to a prospective 7 day design (“in the next 7 days, I intend…”). The intention measures ranged from health behaviours to domestic and academic behaviours. This set of intention items widely represents the types of intentions that current scientific knowledge is based upon (Armitage & Conner, 2001).

### Measures of Intention–Behaviour Relationships

Four measures were computed by dichotomising responses to intentions items into intenders and non‐intenders: (A) Total number of intentions (out of 51), (B) Number of intentions successfully translated, (C) Number of intentions which participants failed to translate,^1^ and (D) Percentage of intentions successfully translated. The deliberate use of dichotomised variables facilitates the communication and illustration of key findings.

The *Mill Hill Vocabulary scale* (Raven, 2000) was used to measure crystallised intelligence. It consists of 88 words divided into two sets. Participants are asked to select the correct synonym for each word from six possible alternatives. The MHV shows good reliability (French & Beaumont, 1990) and validity (Baddeley, Emslie, & Nimmo‐Smith, 1993).

The *Cognitive Failures Questionnaire* (Broadbent, Cooper, FitzGerald, & Parkes, 1982) was used to measure executive action control. The CFQ is a 25‐item questionnaire that enquires about minor mistakes and slips in daily life (“how often do you forget people's names?”). These are often involved in failure to implement intentions. Responses are scaled (0 = never, 4 = always) on three sub‐scales: distraction, memory, and blunders. The CFQ shows good reliability (Bridger, Johnsen, & Brasher, 2013) and validity (Broadbent et al., 1982).

The *DEX questionnaire* (Wilson, Alderman, Burgess, Emslie, & Evans, 1996) is a 20‐item measure which asks about the frequency of behaviours indicative of executive dysfunction in everyday life. It covers the 20 most commonly reported symptoms of frontal lobe dysexecutive syndrome, and samples four areas: (1) emotions, (2) motivation, (3) behaviour, and (4) cognitions. The questionnaire has been validated for use in non‐clinical populations (Chan, 2001). Scores range from 0 to 80, with higher scores indicative of weak executive function.

### Follow‐Up

Forty‐nine behaviours were measured using self‐reports and objective measures of the remaining two (attending a lecture and attending a university sport & recreation facility) were obtained from records linked with student ID numbers.

The *World Health Organization QoL assessment* (WHOQOL; 1998) was used to measure QoL, well‐being, and depression. It has good reliability and validity (WHOQOL, 1998).

Data analyses involved assessing bivariate correlations between individuals’ indicators of intentions (total; succeeded; failed; and percentage implemented) with measures of intelligence, executive function, and quality of life.

## Results Study 1

Participants reported having an average of 18.37 (*SD* = 7.93) intentions out of the 51 possible options for the next week. Out of those, an average of 13 (*SD* = 6.0) were successfully translated and 5.37 (*SD* = 3.75) failed (Table 1).

**Table 1:** Bi‐variate Correlations between Intention–Behaviour Relationships, Intelligence, Executive Function, and Quality of Life

	Intentions	Failed	Succeeded	Percentage
Intentions		.698**	.891**	−.067
Failed			.297**	−.654**
Succeeded				.325**
Percentage				
Sex	.103	−.038	.162	.146
Age	.097	.004	.127	.085
MHVS	.158	−.002	.212*	.176
CFQ total	.076	.091	.044	.023
distraction	.074	.128	.018	−.024
memory	.026	.040	.009	.036
blunders	.102	.068	.093	.065
DEX total	−.132	−.174	−.065	.006
behaviour	−.065	−.120	−.011	.034
cognition	−.047	−.082	−.011	−.045
emotion	−.263**	−.207*	−.219*	−.017
motivation	−.149	−.222*	−.058	.052
QOL rate	.273**	.268**	.194*	−.016
QOL enjoylife	.305**	.227*	.263**	−.016
QOL meaningfullife	.281**	.216*	.238*	.007
QOL selfsatisfaction	.233*	.234*	.162	−.079
QOL depression	−.047	−.081	−.016	.025

Table 1 shows that participants with more intentions experienced both more successful and more failed instances of intention implementation. There was no relationship between the number of intentions held and the percentage rate of success and no indication that those with the highest number of intentions showed a reduced rate of success. Neither total, enacted, nor failed intentions were significantly correlated with age or sex.

As hypothesised, crystallised intelligence (MHV) was significantly correlated with the number of successfully translated intentions and showed a solid null correlation with the number of failures. The relationships between the MHV questionnaire and the number of intentions and the percentage rate of successfully translated intentions were not statistically significant.

Neither of the CFQ subscales showed any relationship to the measures of intention–behaviour relations. The DEX subscale for dysfunctional emotion regulation, however, showed a strong correlation with the number of intentions; people with more problems in regulating their emotions have fewer intentions. The subscale of motivational dysregulation correlated negatively with the number of successfully enacted intentions, but not with the number of total or failed intentions or the percentage success rate of translated intentions.

All WHOQOL measures except for depression showed significant positive relationships with the total number of intentions and, notably, with the number of failed intentions. Three WHOQOL measures showed the highest relationship with the number of intentions successfully translated: overall QoL, enjoying life, and meaningful life. The WHOQOL depression measure did not show any significant correlations with measures of total, successful, and failed intentions, or with the percentage of intentions successfully translated.

## Discussion Study 1

Study 1 found no trade‐off between the number of intentions people form and the rate of successful enactment of intentions. Participants who formed more intentions did not show a lower percentage of successful intention enactment. Indeed, the number of intentions was positively related to QoL and participants with more intentions reported fewer problems with the regulation of emotions. The correlation between crystallised intelligence and successfully enacted intentions suggests that an adaptive pattern of behaviour may be to focus on optimising success, rather than on preventing failure of intention implementation.

While cognitive failure was unrelated to intentionality or to intention–behaviour relationships, people with problems in emotional regulation may avoid forming intentions and experience thereby both fewer failures and successes. In sum, there is no evidence that failed intentions make people miserable. Intending as such is positively related to quality of life.

## Study 2

Health behaviours are often habitual and driven by learned cue‐related automaticity (Verplanken & Faes, 1999). The cognitive ability to *stop and override automatic responses* is therefore of particular interest (Baumeister et al., 1994). Hall et al. (2008) used a Go/No‐Go test (Lapierre, Braun, & Hodgins, 1995) and found both a main effect of executive function as well as an intention‐by‐executive function interaction when predicting dietary and physical activity behaviours. A similar concept, that of *inhibition control*, is measured by the Stroop test (MacLeod, 1991). This measure was found to moderate the intention–behaviour gap for binge drinking and high calorie snacking (Mullan, Wong, Allom, & Pack, 2011) and to predict intention‐incongruent behaviour (Allan et al., 2010). Daly and colleagues (Daly, McMinn, & Allan, 2014) have demonstrated that *cognitive flexibility* (measured with a word fluency task) predicts engagement in physical activity behaviour over time. Study 2 tested the relationship between objectively measured executive functioning (both cognitive inhibition and cognitive flexibility) and intra‐individual measures of intention–behaviour consistency in a small pilot study.

## Methods Study 2

An All‐Intentions Method (AIM) similar to Study 1 was conducted with 30 university students (mean age of 23.9, *SD* = 8.19, 12 were women). In addition to the measures of intention and behaviour, a lab‐based assessment was conducted to collect the measures of executive function:

A *Go/NoGo* computer task was used to measure cognitive inhibition. Participants were required to regulate their responses to visually presented stimuli and decide either to initiate responses quickly or inhibit response. This is a computer‐administrated visual Go/NoGo paradigm modelled after LaPierre et al. (1995). Participants were asked to press the space bar as quickly as possible whenever they saw a capital letter on the screen and to press nothing (i.e. to inhibit their response) when a small letter appeared. After a practice block, participants completed the two Go/NoGo blocks A and B (A where Go trials were more frequent and B where NoGo trials were more frequent). The order of these two blocks was randomly varied. Scoring was based on totalling commission errors (incorrectly responding to a *no‐go* cue) and omission errors (not responding to a *go* cue) over the two 100‐trial blocks, and on choice reaction time (ms).

*Stroop test*. A computerised version of the Stroop test (Trenerry, Crosson, DeBoe, & Leber, 1989) was used as a second measure of cognitive inhibition. Participants were asked to first name the colour of a series of non‐words (“XXXs”) in a practice session at the start of the test. They were then shown a series of colour words (e.g. “blue”, “red”) printed in a colour of ink different from the colour name they represent (e.g. the word “blue” printed in red ink). Participants must name the colour that the incongruently coloured test words are printed in as quickly as possible. Participants were scored according to their accuracy and time.

The *Word fluency* test requires participants to rapidly produce a list of words each containing a specific letter that has been previously specified by the examiner, in two blocks of 2 minutes each after a practice trial (Guilford & Guilford, 1980). A numerical measurement was derived from the number of correct words generated using the letters “B” then the letter “T” without using places, names, or three‐letter words. Word fluency provides a measure of cognitive flexibility, a key component of executive functioning.

Each study measure was correlated with the individual correlation coefficient between intentions and behaviours for each participant (individual intention behaviour congruence).

## Results Study 2

The individual intention–behaviour congruence was not related to Go/NoGo speed in either block A (*r* = .13; *p *=* *.25) or B (*r *=* *.221; *p *=* *.13) or to the Stroop effect (*r *=* *.05; *p *=* *.40). However, the intention–behaviour congruence was related to the errors made during both cognitive tasks, *r *=* *.58; *p *<* *.001 for the Go/NoGo error and *r *=* *.35; *p *=* *.028 for Stroop error. The word fluency task correlated significantly with individual intention–behaviour congruence *(r *=* *.34; *p *=* *.035).

## Discussion Study 2

Verbal fluency as well as errors in task switching and inhibition control are related to individual intention–behaviour congruence. Given the limited sample size, it cannot be ruled out that there are other meaningful relationships that remained undetected; however, these are unlikely to be very large.

## Overall Discussion

This paper introduced a new paradigm for investigating intention–behaviour relationships based on measures of multiple intentions and behaviours. This approach allows researchers to disentangle how many intentions a person holds, how many they successfully implement, and how many they fail to implement. This enables a more detailed analysis of how intentions and behaviours relate to each other and how this relationship reflects other personal features. By generating measures of intention–behaviour relationships for each individual, this new approach can then in turn be used as a person‐specific indicator for further inquiry.

Two studies provided a proof of principle of how this approach can be usefully applied. Study 1 demonstrated that the number of intentions participants formed was not related to the percentage of intentions successfully enacted. This is a novel finding, countering the common‐sense assumption that forming too many intentions may set oneself up for failure. Reported cognitive failures, behavioural and cognitive dysfunctions in everyday life were not related to intentions and intention–behaviour relationships. This finding is in contrast to previous evidence from a single‐behaviour study where DEX scores explained variance in the intention–behaviour gap for fruit and vegetable consumption (Allan et al., 2011). The relationship between crystallised intelligence and the number of successfully enacted intentions is a finding which is novel and requires replication. It suggests that crystallised intelligence might be specifically related to goal setting strategies that enable success, rather than strategies that avoid failure. Individuals who reported difficulties with emotion control reported fewer intentions, but there was no relationship to the percentage of intentions implemented. Individuals who reported difficulties with the control of their motivation reported fewer failed intentions.

A strong positive relationship was found between QoL and intentions regardless of their implementation. These findings are incongruent with the idea that unfulfilled intentions always have costs. This might be the case under certain circumstances, but further theorising and research is needed to identify those circumstances, ideally in a multiple‐behaviour paradigm.

Because of the mostly self‐reported nature of the cognitive measures in Study 1, Study 2 was conducted to test for relationships between objective measures of executive function and individual intention–behaviour congruence. Study 2 suggests that for inhibition control tasks, error rates (indicative of actual failures of inhibitory control), rather than overall performance (indicative of both general speed/efficiency and failures), were strongly related to the intention–behaviour congruence of individuals. Moreover, the measure of cognitive flexibility (word fluency task) also showed a significant relationship to participants’ ability to act in line with their intentions.

The two studies reported here were conducted to provide an initial proof of principle for the main ideas underlying the multiple intention approach. These studies illustrated the application of this approach and suggested novel findings. There were clear limitations in both studies which we would like to highlight. They include the self‐reported predictive measures in Study 1, the small sample size in Study 2, the use of convenience student samples in both studies, and the limited follow‐up period of only a week. Based on these limitations, the main study findings require replication. Likewise, it would be feasible to conduct more sophisticated statistical analyses over the data, but the intention of this paper is to describe and illustrate the novelty of the approach, Given the limitations listed above, a more complex statistical analytic approach was not seen as desirable.

### Future Research

The aim of the present research was to provide a simple and transparent illustration of the use of the All‐Intentions Method (AIM) in a multiple‐behaviour paradigm. The paradigm of assessing various intentions (potentially alongside other predictors) and various behavioural outcomes simultaneously (potentially over various time points) would allow more complex multi‐level analyses of datasets addressing more complex hypotheses. Intentions and behaviours are not independent of each other and it is possible to aggregate them empirically using factor analytical methods, potentially following taxonomies of behaviours (McEachan, Lawton, & Conner, 2010; Nudelman & Shiloh, 2015) or conceptually by contrasting intentions for behaviours with known interrelationships such as physically active and sedentary behaviours (Rhodes & Blanchard, 2008). Alternatively or in addition to this approach, participants may be asked how they actually perceive the interrelation of their multiple goals and/or behaviours. This would allow a rigorous investigation of the role of cross‐behaviour cognitions (Rhodes & Blanchard, 2008) such as goal conflict, goal facilitation, and goal priority (Conner et al., in press; Gebhardt & Maes, 1998; Presseau et al., 2010; Presseau et al., 2013; Riediger & Freund, 2004) as well as transfer cognitions and compensatory health beliefs (Berli, Loretini, Radtke, Hornung, & Scholz, 2014; Fleig et al., 2015; Knäuper, Rabiau, Cohen, & Patriciu, 2004; Nigg, Lee, Hubbard, & Min‐Sun, 2009).

In the present research the role of a limited number of cognitive moderators of the intention–behaviour relationship was investigated. The new paradigm offers the opportunity to test a range of personality, environmental, social cognitive, habitual, and self‐regulatory moderators of cognition–behaviour relationships more rigorously in a multiple‐behaviour context (de Bruijn, Brug, & van Lenthe, 2009; de Bruijn et al., 2007; Kothe, Sainsbury, Smith, & Mullan, 2015; Presseau et al., 2014; Rhodes & Dickau, 2013; Skår, Sniehotta, Araújo‐Soares, & Molloy, 2008).

Finally, future research investigating the mechanisms of behaviour change can be enhanced in particular by evaluating interventions in a multiple‐behaviour paradigm. Fleig and colleagues demonstrated that an exercise intervention which was found to be effective in increasing physical activity and exercise habit strength also had a secondary effect on fruit and vegetable intake, and that this secondary effect was moderated by changes in exercise habit strength (Fleig, Lippke, Pomp, & Schwarzer, 2011). These findings may be interpreted as an indication that the increased automaticity associated with the exercise habit has freed self‐regulatory capacity that participants could use to more effectively regulate other desirable health behaviours. This is also the hypothesised mechanism behind many interventions involving the formulation of one or more action plans (or implementation intentions) which are hypothesised to facilitate behaviour change by increasing the automaticity of behavioural performance (de Bruijn, Wiedemann, & Rhodes, 2014; Sheeran, Webb, & Gollwitzer, 2005; Verhoeven, Adriaanse, de Ridder, de Vet, & Fennis, 2013; Wiedemann, Lippke, & Schwarzer, 2012). Evaluated in a multiple‐behaviour paradigm, the known mechanisms of planning interventions can be contrasted against a competing hypothesis, that planning increases the salience and priority of certain behaviours over that of alternative options. In this case, the success of a self‐regulatory intervention might also have hidden costs for competing intentions that are not traditionally measured in evaluation studies.

In conclusion, the proposed multiple‐behaviour approach provides an opportunity to examine the relationship between the number of intentions and their enactment and to test novel hypotheses about intention–behaviour relationships, their predictors, and consequences. It may contribute to the understanding of intention–behaviour relationships in addition to traditional single‐intention studies.

## Supporting information


**Appendix S1:** Assessment of intentions

## Note

1 As the number of intentions varies between participants, the number of failed and the number of successfully implemented intentions are conceptually different.
