# Supplementary material for: “You Can't Always Get What You Want”: A Novel Research Paradigm to Explore the Relationship between Multiple Intentions and Behaviours
Source: Appl Psychol Health Well Being. 2016 May 27;8(2):258–75. doi: 10.1111/aphw.12071 (PMC4949534; doi:10.1111/aphw.12071)
Supplement: Supplementary file 1 — Appendix S1: Assessment of intentions [file APHW-8-258-s001.docx]

Appendix 1: Assessment of intentions

Over the next seven days, I intend to ….

1. ... donate blood
2. ... take vitamin pills
3. ... eat meat
4. ... use dental floss
5. ... tidy my room/flat
6. ... go to the library to study
7. ... go for a walk
8. ... eat fast food
9. ... buy a gift for a friend
10. ... read for pleasure
11. ... recycle my household newspapers and other papers
12. ... eat breakfast every day
13. ... get drunk
14. ... buy a magazine
15. ... go to a nightclub
16. ... perform self-examinations of my breast (for women)/testicles (for men)
17. ... write 2 letters/emails to family/friends
18. ... use Ecstasy
19. ... go shopping with a friend
20. ... buy a newspaper
21. ... recycle my bottles
22. ... smoke cigarettes
23. ... eat vegetables
24. ... exercise
25. ... go to the pub
26. ... attend all my lectures
27. ... visit the countryside
28. ... eat salads
29. ... buy new clothes
30. ... eat chips
31. ... go to the cinema
32. ... engage in vigorous physical activity for at least 30 minutes
33. ... go out for a meal
34. ... climb a hill or mountain
35. ... engage in moderate physical activity for at least 30 minutes
36. ... have sexual intercourse
37. ... get at least 7 hours sleep on the five weekdays
38. ... go jogging
39. ... participate in vigorous physical activity
40. ... use a condom when having sexual intercourse with a new partner
41. ... go home to visit your parents
42. ... use the University’s Sport and Recreation Facility
43. ... visit friends
44. ... drink little or no alcohol
45. ... avoid calories in drinks and snacks between meals
46. ... sleep in past 9:00 a.m. on the weekdays
47. ... eat five portions of fruit and vegetable
48. ... eat cakes and biscuits
49. ... follow a low-fat diet
50. ... join the University’s Conservation Society
51. ...attend your [Health Psychology] lecture (dependent on lecture from which students were recruited)

Measures of behaviour were assessed seven days later asking: Over the last seven days, have you …. (using the same behaviours as listed above.
